# Variability in Cognitive Performance on Mobile Devices Is Sensitive to Mild Cognitive Impairment: Results From the Einstein Aging Study

**DOI:** 10.3389/fdgth.2021.758031

**Published:** 2021-12-03

**Authors:** Eric S. Cerino, Mindy J. Katz, Cuiling Wang, Jiyue Qin, Qi Gao, Jinshil Hyun, Jonathan G. Hakun, Nelson A. Roque, Carol A. Derby, Richard B. Lipton, Martin J. Sliwinski

**Affiliations:** ^1^Department of Psychological Sciences, College of Social and Behavioral Sciences, Northern Arizona University, Flagstaff, AZ, United States; ^2^Center for Healthy Aging, College of Health and Human Development, Pennsylvania State University, University Park, PA, United States; ^3^Department of Neurology, Albert Einstein College of Medicine, Bronx, NY, United States; ^4^Department of Epidemiology and Population Health, Albert Einstein College of Medicine, Bronx, NY, United States; ^5^Department of Neurology, College of Medicine, Pennsylvania State University, Hershey, PA, United States; ^6^Department of Psychology, University of Central Florida, Orlando, FL, United States

**Keywords:** cognitive performance variability, cognitive health, mild cognitive impairment–MCI, mobile cognitive assessment, technology, ecological momentary assessment (EMA), intraindividual variability (IIV), Alzheimer's disease and related dementias (ADRD)

## Abstract

**Background and Objective:** Within-person variability in cognitive performance has emerged as a promising indicator of cognitive health with potential to distinguish normative and pathological cognitive aging. We use a smartphone-based digital health approach with ecological momentary assessments (EMA) to examine differences in variability in performance among older adults with mild cognitive impairment (MCI) and those who were cognitively unimpaired (CU).

**Method:** A sample of 311 systematically recruited, community-dwelling older adults from the Einstein Aging Study (Mean age = 77.46 years, SD = 4.86, Range = 70–90; 67% Female; 45% Non-Hispanic White, 40% Non-Hispanic Black) completed neuropsychological testing, neurological assessments, and self-reported questionnaires. One hundred individuals met Jak/Bondi criteria for MCI. All participants performed mobile cognitive tests of processing speed, visual short-term memory binding, and spatial working memory on a smartphone device up to six times daily for 16 days, yielding up to 96 assessments per person. We employed heterogeneous variance multilevel models using log-linear prediction of residual variance to simultaneously assess cognitive status differences in mean performance, within-day variability, and day-to-day variability. We further tested whether these differences were robust to the influence of environmental contexts under which assessments were performed.

**Results:** Individuals with MCI exhibited greater within-day variability than those who were CU on ambulatory assessments that measure processing speed (*p* < 0.001) and visual short-term memory binding (*p* < 0.001) performance but not spatial working memory. Cognitive status differences in day-to-day variability were present only for the measure of processing speed. Associations between cognitive status and within-day variability in performance were robust to adjustment for sociodemographic and contextual variables.

**Conclusion:** Our smartphone-based digital health approach facilitates the ambulatory assessment of cognitive performance in older adults and the capacity to differentiate individuals with MCI from those who were CU. Results suggest variability in mobile cognitive performance is sensitive to MCI and exhibits dissociative patterns by timescale and cognitive domain. Variability in processing speed and visual short-term memory binding performance may provide specific detection of MCI. The 16-day smartphone-based EMA measurement burst offers novel opportunity to leverage digital technology to measure performance variability across frequent assessments for studying cognitive health and identifying early clinical manifestations of cognitive impairment.

## Introduction

Alzheimer's disease is the sixth leading cause of death in America, impeding health and well-being for an estimated 6.2 million Americans currently living with the dementia stages of the illness (1). Alzheimer's disease and related dementias (ADRD) also impact caregivers, families, and communities responsible for over 15 billion h of unpaid care and $350 billion in annual health care costs (1). Older adults with mild cognitive impairment (MCI), conceptualized as an intermediary stage between normative cognitive aging and dementia, have more memory and thinking problems than their age and education-level peers (2, 3). Individuals with MCI may be targeted for secondary prevention efforts. The present study describes a digital approach to the measurement of ebbs and flows in cognitive performance in everyday life through intensive repeated measurement. Specifically, we assess variability in test performance on mobile devices that may inform subtle differences in everyday cognitive performance between individuals with MCI and those who were cognitively unimpaired (CU).

Conventional approaches to distinguishing cognitive impairment typically involve evaluating the *level* of cognitive performance using single administrations of one or several cognitive tests [e.g., (4, 5)]. This approach ignores within-person variability in cognitive performance–a promising behavioral signature with potential to distinguish normative and pathological cognitive aging [e.g., (6, 7)]. Further, MCI is an unstable marker of cognitive pathology; recent reports and meta-analyses show that from 14 to 58% of individuals classified as MCI revert to being CU at a follow-up evaluation [e.g., (8–11)]. Several factors may contribute to reversion. First, cut-scores used to define MCI are usually developed solely based on the distribution of scores in the CU group, without considering the distribution of scores among individuals with impairment; we have recommended the use of diagnostic norms that differentiate groups rather than comparative norms which consider only the distribution of scores in the CU group (12). Second, the single shot cognitive assessments used to diagnose MCI have modest reliabilities resulting in random measurement error that may contribute to misclassification. Third, cognitive performance systematically varies within person in daily life, often in relation to known risk factors [e.g., (13–15)]. Indeed, variability in cognitive performance within a day and across days is theorized to reflect, at least in part, environmental influences and psychosocial processes that also vary throughout the day and from day-to-day (16, 17). In this context, reversion may result from accurate measurement of MCI status that falls below a cut-score at the time of the assessment on a bad day. “Bad day” effects may lead to false positive MCI classification. “Good day” effects may lead to false negative classification. We hypothesize that individuals with MCI exhibit greater variability in cognitive performance; the variability in performance in everyday life, then, could contribute to unreliability in diagnosis and reversion.

Measuring cognitive function with ecological momentary assessments (EMA) is ideal for monitoring these patterns of variability from assessment-to-assessment within a day and from day-to-day. In the present study, we use a smartphone-based digital health approach with EMA to address these issues by repeatedly measuring cognitive performance in people's natural environments, multiple times per day, across multiple days. Using this digital health approach, we aim to identify those at increased risk of MCI by examining not just level of performance, but variability in performance. We also control for the role of everyday contexts that may impact performance variation within and across study days.

### Variability in Cognitive Performance as an Indicator of Cognitive Impairment

Over the past 20 years, research shows that adults with cognitive impairment are more variable in their cognitive test performance [e.g., (6, 7, 18)], with the majority of this research measuring variability across trials within a testing session [e.g., (19–21)]. Trial-level variability has been associated with cognitive decline [e.g., (22)], mild impairments [i.e., MCI classification, (6); cognitively impaired-not-dementia (CIND) classifications, (7)], and dementia [e.g., (18)], identifying its potential utility as an important behavioral indicator of cognitive impairment. Indeed, greater trial-level variability has been associated with neurological mechanisms of cognitive impairment such as decreases in dopamine activity measured by positron emission tomography (23) and other systematic changes to central nervous system integrity [e.g., white matter hyperintensities (24, 25)]. Performance variability across timescales longer than trial-to-trial fluctuations, however, are relatively untapped as indices useful in the measurement of cognitive impairment in everyday life.

Measuring individuals multiple times per day across multiple days unlocks the capacity to assess performance variability across longer timescales than the trial-level. Recent advancements in digital technology can be leveraged for this pursuit by using ecological momentary assessment (EMA) designs [e.g., (26–28)]. EMA features frequent, brief assessments of psychosocial health and behavior in naturalistic settings. EMA protocols that embed ultra-brief mobile cognitive tests facilitate measurement of multiple “snapshots” of an individual's cognitive performance both within study days (i.e., within-day variability across assessments) as well as across days (i.e., day-to-day variability).

Characterizing both timescales of within-day variability and day-to-day variability will inform future digital health approaches. For example, determining the differences in within-day and day-to-day variability in performance between individuals with MCI and those who are CU could inform future data collection strategies. Schmiedek et al. (29) showed reliable, systematic day-to-day variability that differed across age groups (i.e., older adults exhibited less day-to-day variability than younger adults) and across cognitive domains (i.e., greater proportions of day-to-day variability were found in perceptual tasks compared to working memory and episodic memory tasks). It may be that older adults exhibited less day-to-day variability in cognitive performance than younger adults because older participants lead more routinized lives than younger participants and thus had fewer differences in context to drive ebbs and flows in performance. Measuring both cognitive performance multiple times per day along with information on context before and during performance in naturalistic settings will facilitate an understanding of variability and the contextual factors that contribute to it. The present study extends this analysis of single assessments completed each day in a research office by evaluating cognitive status (0 = CU, 1 = MCI) differences in the variability in mobile cognitive performance both within-day *and* from day-to-day in everyday life.

EMA introduces naturalistic and uncontrolled settings to the digital measurement of cognitive performance. While context factors such as distractions, who is around you while completing a test, and where you are located are commonly controlled for in a laboratory setting (e.g., individual testing sessions in a quiet research office with minimized external distractions), these factors are key aspects of EMA designs that take place across a variety of physical and social settings. Contextual factors such as distractions, social company, and location may influence variability in cognitive performance and obfuscate variability's sensitivity to cognitive impairment. The present study formally tests whether possible relationships between cognitive status and variability in mobile cognitive performance are driven by environmental context by controlling for the influence of distraction, social company, and location.

#### Digital Health Approach

Advances in smartphone-based digital health approaches with mobile cognitive tests have enabled reliable and valid repeated assessments of cognitive function in naturalistic settings (26–28). Earlier work by our team has shown the feasibility of integrating mobile cognitive assessments in an EMA protocol among a diverse adult lifespan sample (ages 25–65) in the Bronx, NY (27). In the Effects of Stress on Cognitive Aging, Physiology and Emotion [ESCAPE; (30)] project, participants completed smartphone-based cognitive tests up to 5 times per day for 14 consecutive days. Reliability for average scores on these mobile cognitive tests of working memory (Grid Memory) and processing speed (Symbol Match) ranged from 0.97 to 0.98. Both tasks demonstrated construct validity with factor loadings exceeding 0.60 on relevant cognitive constructs and criterion validity with significant correlations between in-lab assessments and Symbol Match and Grid Memory (rs ranged from −0.39 to −0.45). High adherence, reliability, and validity of cognitive assessments in adults within the ESCAPE study informed the extension of these digital health approaches to a sample over the age of 70 in the Einstein Aging Study (EAS). In the present study we leverage the Mobile Monitoring of Cognitive Change (M2C2) platform, an innovative mobile platform funded by the NIH for EMA-based delivery of ultra-brief mobile cognitive testing. M2C2, currently in the development, validation, and norming phase [e.g., (27, 31)], enables our digital health approach to assess cognitive performance under naturalistic circumstances multiple times per day, across multiple days.

Past research has found greater trial-level variability in laboratory settings to be a promising indicator of cognitive impairment [e.g., (7)]. Critical gaps in this literature, however, include the open questions of whether estimates of variability operationalized at longer timescales than the trial-level and obtained via mobile devices distinguish individuals with MCI from individuals who are CU. To our knowledge, the present study is the first EMA study to explore the potential utility of variability in mobile cognitive performance within-day and across days as sensitive markers to distinguish MCI from CU.

#### Within-Day vs. Day-to-Day Variability Timescales

Evaluating whether cognitive status differences emerge at both within-day and day-to-day variability timescales (or if differences emerge in one timescale but not the other) has important implications for the design of future digital health approaches for intensive repeated measurement of cognitive performance. For example, if cognitive status differences emerge in within-day variability, but not in day-to-day variability, studies interested in quantifying variability in cognitive performance sensitive to MCI should prioritize collecting more assessments within a day rather than across days. We simultaneously assess cognitive status differences in within-day variability and day-to-day variability in three domains of cognitive function.

#### Multiple Domains of Cognitive Performance

Most research on variability in cognitive performance focuses on processing speed [e.g., (20)]. Cognitive variability in other domains of cognitive performance remain to be explored. Tasks requiring memory binding, for example, are sensitive to Alzheimer's disease (32–34), whereas less is known about the sensitivity of tasks requiring spatial working memory to pathological cognitive aging outcomes. We evaluate three domains of cognitive function in the present study: processing speed, visual short-term memory binding, and spatial working memory.

### The Present Study

#### Research Questions

To address these gaps in the understanding of variability in EMA cognitive performance in relation to cognitive status (0 = CU, 1 = MCI), we use baseline data from the EAS, a longitudinal study of community dwelling older adults in Bronx County, NY. Past research suggests that individuals with MCI perform worse on mobile cognitive tests in terms of level of performance [e.g., (35)]. The purpose of the current study is to determine whether individuals with MCI also exhibit greater variability in performance within intensive repeated measurements within and across study days. We ask two research questions.

First, does variability in performance on mobile cognitive tests differ for individuals with MCI and those who are CU? We hypothesize individuals with MCI will exhibit greater variability in performance compared to those who were CU, where MCI is defined independent of performance on the mobile cognitive tests. For each mobile cognitive test, we evaluate cognitive status (0 = CU, 1 = MCI) differences in the variability in mobile cognitive performance across sessions within a day as well as across study days to examine if differences in variability emerge at the within-day level vs. the across-day-level timescales or both. We further examine whether findings differ across cognitive domains to evaluate whether mobile tests requiring speeded responses or memory binding are particularly sensitive to MCI based on past research on trial-level variability's established sensitivity to MCI and performance on memory binding tasks being sensitive to ADRD (32, 33, 36). The extent to which cognitive status is related to performance variability in mobile cognitive tests requiring spatial working memory compared to speeded responses and memory binding were evaluated in exploratory fashion due to limited prior research attention.

Addressing research question 1 will clarify if there are significant differences in the variability in mobile cognitive test performance across sessions within a day and across study days between the MCI and CU groups. However, it does not account for the potential influence of contextual factors. Differences in participants' contexts before and during completion of the cognitive tests may relate to their performance on the tests. To formally test these possible confounders, the second research question asks to what extent is cognitive status related to variability in mobile cognitive performance after controlling for EMA contextual factors? We test the effects of distraction, location, and social company as key contextual factors that may influence the relationship of cognitive status with cognitive variability.

## Materials and Methods

### Study Design and Procedure

**Figure 1:**
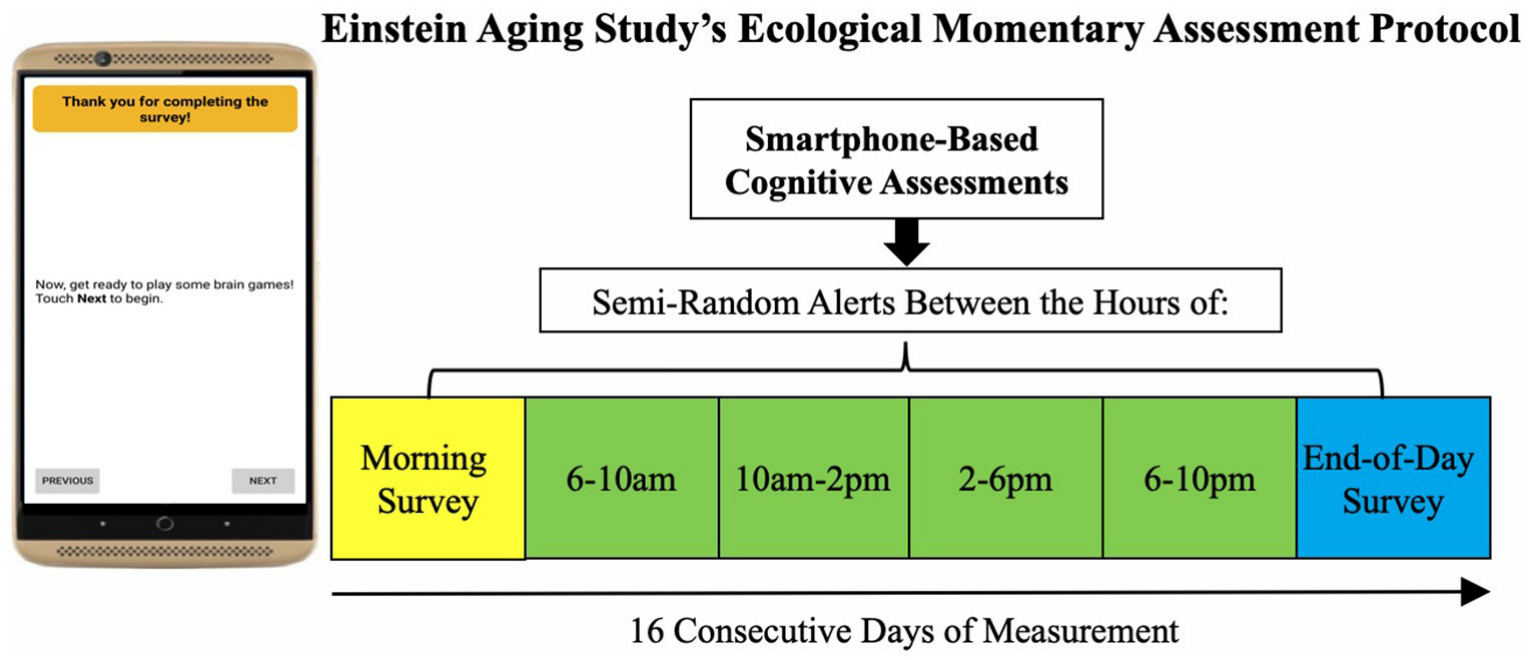
Study design for EAS digital health approach.

We utilized baseline data from the Einstein Aging Study (EAS), a longitudinal study of early detection of cognitive impairment in community-dwelling older adults from Bronx County, NY. Recruitment involved systematic sampling from registered voter lists in the Bronx, NY. Exclusion criteria for enrollment included: age younger than 70 years; baseline diagnosis of dementia defined by application of standard criteria (37); alcohol/substance abuse within the past 6 months; sensory, motor, or other conditions that may interfere with participation; chemotherapy within the past year. Following recruitment and obtaining informed consent, participants visited the EAS clinic to complete a full battery of neuropsychological testing and neurological assessment. Participants attended a second clinic visit at which they were trained to complete the EMA protocol of self-report items followed by brief cognitive tests on study-provided smartphone mobile devices. Participants completed a self-initiated morning and end-of-day survey (2–3 min each/survey) and four semi-random beeped assessment occasions during the day (4–5 min each/day), taking about 20–25 min total/day (Figure 1). Each participant was asked to complete up to 96 measurement occasions across 16 days (16 morning, 64 beeped, 16 end-of-day assessments).

### Sample Demographics

The sample consisted of 311 systematically recruited community-dwelling older adults (Mean age = 77, SD = 5, Range = 70–90) from Bronx County, NY, a racially and ethnically diverse urban setting with a population of 1.4 million [11.8% older adults; (38)]. Women comprised 67% of the sample, and average years of education was 15 years (SD = 3.6). The sample was 45% Non-Hispanic White, 40% Non-Hispanic Black, and 15% other races/ethnicities (10% Hispanic White, 3% Hispanic Black, 1% Asian, <1% other, <1% more than one race). Of the 311 recruited participants, 100 were classified with MCI and 211 were CU.

### Cognitive Status: Mild Cognitive Impairment and Cognitively Unimpaired Classifications

Participants were classified as having MCI or being CU based on the Jak/Bondi algorithmic criteria (2, 39) of global neuropsychological test performance in the EAS clinic [see (40) for additional information]. The following 10 neuropsychological instruments covering five cognitive domains were considered for this classification: (1) Memory: Free recall from the Free and Cued Selective Reminding Test (41), Benson Complex Figure [Delayed (42)]; (2) Executive Function: Trail Making Test Part B [limit time 300 s (43)], Phonemic Verbal Fluency [Letters F, and L for 1 min each (44)]; (3) Attention: Trail Making Test Part A [limit 150 s (43)], Number Span [forward and backward (45)]; (4) Language: Multilingual Naming Test [MINT, total score (46)], Category Fluency [Animals, Vegetables: 1 min each (47)]; (5) Visual-spatial: Benson Complex Figure [Immediate (42)], WAIS III Block Design (45). The following actuarial formula was used: (1) impaired scores, defined as >1 SD below the age, gender, and education adjusted normative means, on both measures within at least one cognitive domain (i.e., memory, language, or speed/executive function); or (2) one impaired score, defined as >1 SD below the age, gender, and education adjusted normative mean, in each of three of the five cognitive domains measured; or (3) a score of 4 on the Lawton Brody scale, indicating dependency on all four instrumental activities items (48). Otherwise, an individual was classified as being CU.

### Mobile Cognitive Tests

**Figure 2:**
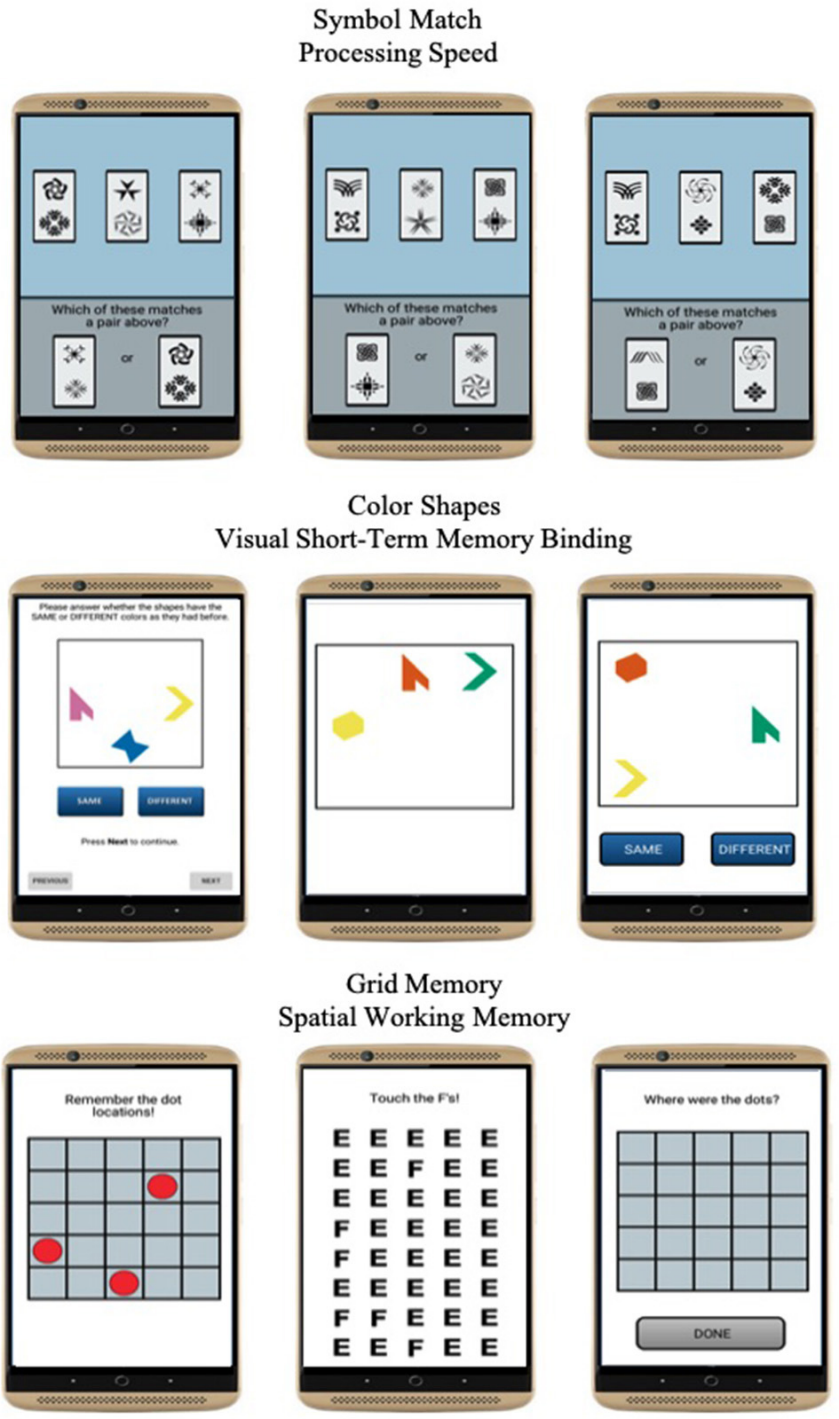
Screenshots of mobile cognitive tests.

Three mobile cognitive tests were administered to evaluate processing speed, visual short-term memory binding, and spatial working memory domains of cognitive function (Figure 2). Each test took approximately 45 s to complete on average.

#### Processing Speed

Symbol Match was used to measure processing speed (27). Participants were asked to compare three symbol pairs at the top of the screen with two symbol pairs at the bottom of the screen and decide as quickly and accurately as possible which of the bottom-screen pairs matches a top-screen pair. The task comprised of 11 trials. Mean response time of correct trials was used to operationalize performance. Higher values reflected slower processing speed.

#### Visual Short-Term Memory Binding

Color Shapes is a change detection paradigm used to measure visual short-term memory binding (34, 36). Participants are asked to memorize the shapes and colors of three different polygons for 3 s. The three polygons are then removed from the screen and re-displayed at different locations, either having the same or different colors. Participants are then asked to decide whether the combination of colors and shapes are the “Same” or “Different” between the study and test phases. The task comprised of 5 trials. A corrected recognition score calculated as hits (# of correct “Different” responses)–false alarms (# of incorrect “Different” responses) was used to operationalize performance and correct for the potential of selecting the “Same” response at every trial. Higher values reflected better binding performance.

#### Spatial Working Memory

Grid Memory was used to measure spatial working memory (27). In an initial brief study phase, participants are asked to memorize the location of 3 dots presented at random locations on a 5 x 5 grid for 3 s. After an 8-s letter-cancellation visual distractor phase, participants are then asked to recall the location of each dot during the study phase. The free recall phase requires participants to touch the locations in an empty 5 x 5 grid in which the 3 dots were initially presented. The task comprised of 2 trials. A Euclidean distance error score giving partial credit based on the deviation of recalled locations compared to correct locations was used to operationalize performance. Higher values reflected worse spatial working memory performance.

### EMA Contextual Variables

Three time-varying binary contextual variables that could influence variability in cognitive performance were examined: location, social company, and distraction. Prior to the mobile cognitive tests, single items were asked pertaining to the participants' current location (at home vs. elsewhere) and social company (alone vs. with others). Following the mobile cognitive tests, a single item was asked pertaining to whether anything distracted them while performing the mobile cognitive tests (distracted vs. undistracted).

### Operationalizing Mean and Variability in Cognitive Performance

Heterogenous variance multilevel models (HV-MLM) were used to simultaneously model mean and variability [e.g., (49–51)]. Within the HV-MLM, mean performance was modeled with fixed effects and variability in performance was modeled using random effects at person and day levels with variance allowed to depend on cognitive status, and with log-linear prediction of the residual variance ([see Equation (1) below]. This computationally efficient approach is suitable for determining correlates of variability while accounting for mean performance and serves as our operationalizations of both mean performance and variability in cognitive performance across assessments. Specific parameterization and details on log-linear prediction of the residual variance are provided below.

### Adherence to EMA Protocol

**Figure 3:**
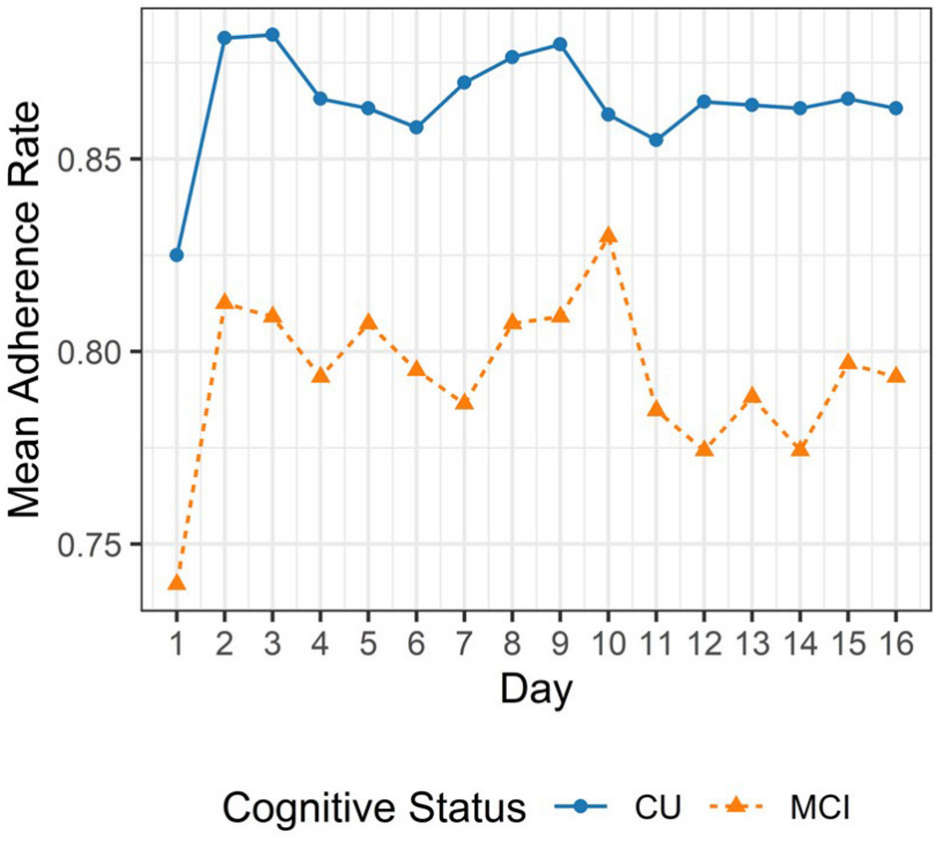
Mean adherence to EMA protocol.

Adherence rate for each participant was calculated as the proportion of complete EMA sessions out of 96 sessions. A complete EMA session was defined as a session where the participant completed 11 trials, 2 trials, and 5 trials for the Symbol Match, Grid Memory, and Color Shapes tests, respectively. The analytic sample comprised of 24,755 complete mobile cognitive assessments. We excluded 341 EMA sessions (<1.5% of available EMA sessions) due to incompleteness. Median adherence rate to the 16-day EMA protocol was 82% for individuals with MCI (Interquartile Range: 68–92%) and 90% for individuals who were CU (Interquartile Range: 79–95%). Figure 3 provides a line plot displaying the mean adherence rate within each day across the 16-day EMA protocol.

### Analytic Strategy

We utilized multilevel modeling [PROC MIXED (52)]] given the three-level nested structure of the data (i.e., up to 6 assessments nested within up to 16 days nested within 311 participants; yielding up to 96 assessments per person). To first understand the variance decomposition in mobile cognitive test performance, unconditional three-level linear mixed models were used to estimate the proportion of total variance attributable to each of the three components: between-person, within-person across days, and within-person within days. To address the two research questions, we utilized three-level heterogenous variance multilevel models (HV-MLM). Equation (1) below expresses the heterogeneous variance modeling approach that predicts short-term variability in mobile cognitive test performance for individual *i* on day *d* at assessment session *s* as a function of cognitive status (0 = CU, 1 = MCI). For Research Question 1, we controlled for linear and quadratic trends across assessment sessions and study days to account for practice-related/learning effects [e.g., (53)]. Preliminary analyses examining potential cognitive status differences in these practice-related/learning curves indicated that the linear and quadratic trends did not vary as a function of cognitive status (*p*s > 0.05). As such, interaction terms of cognitive status with the linear and quadratic trends across sessions and days were dropped from analyses. For Research Question 2, we further controlled for sociodemographic and contextual variables. Sociodemographic covariates included grand-mean-centered age and years of education (centered at the sample averages to facilitate meaningful interpretations of intercept and slope parameter estimates), as well as sex and race/ethnicity. Dichotomous variables were used to capture three time-varying EMA contextual influences on variability in cognitive performance: location (at home vs. elsewhere), social company (alone vs. with others), and distraction (distracted vs. undistracted). To separate within-person and between-person effects, person-mean scores on each variable (i.e., proportion of occasions on which a participant was at home, alone, and/or distracted; “mn” appended to variable names in equation below) were included along with the time-varying dichotomous context variables.

**Table:** 

HV-MLMs for Mean and Variability in Mobile Cognitive Test Performance (1)	
Level-1 (session):	CognitiveTestPerformance*_*sdi*_* = *β_0*di*_*(Intercept) + β_1_(Session*_*sdi*_*) + β_2_(Session_sdi_*Session*_*sdi*_*) + β_3_(Distraction*_*sdi*_*) + β_4_(Location*_*sdi*_*) + β_5_(SocialCompany*_*sdi*_*) + e*_*sdi*_*
Variance of residual:	Var(e*_*sdi*_*) = exp[*z*_0_(Intercept*)*+ *z*_1_(Session*_*sdi*_)* + *z*_2_(Session_sdi_*Session*_*sdi*_)* + *z*_3_(Day_di)+*z*_4_(Day_di_*Day_di)+*z*_5_(CognitiveStatus*_*i*_*) +*z*_6_(Age_i)+*z*_7_(Education_i)+*z*_8_(Sex_i)+*z*_9_(Race/Ethnicity_i)+*z*_10_(Distraction*_*sdi*_)* + *z*_11_(Distractionmn_i)+*z*_12_(Location_sdi)+*z*_13_(Locationmn_i)+*z*_14_(SocialCompany_sdi)+*z*_15_(SocialCompanymn*_*i*_)*]
Level-2 (day):	β_0di_ = δ_00i_ + δ_01_(Day*_*di*_*) + δ_02_(Day_di_*Day*_*di*_*) + *U*_0di_
Level-3 (person):	δ_00i_ = γ_000_ + γ_001_(CognitiveStatus_i_) + γ_002_(Age_i_) + γ_003_(Education_i_) + γ_004_(Sex_i_) + γ_005_(Race/Ethnicity_i_) + γ_006_(Distractionmn_i_) + γ_007_(Locationmn_i_) + γ_008_(SocialCompanymn_i_) + *V*_00i_

Research Question 1 examined potential cognitive status differences in variability in mobile cognitive test performance across assessment sessions (*z*_5_). Research Question 2 required additional within-person and between-person sociodemographic and EMA contextual terms to be added to the model to assess the extent to which a possible relationship between cognitive status and variability in cognitive performance is robust to the influence of EMA context. At level-1 (session-level), cognitive performance for person *i* on day *d* at assessment session *s* was defined as a function of an intercept (β_0di_), linear (β_1_), and quadratic (β_2_) trends across sessions, within-person distraction (β_3_), within-person location (β_4_), within-person social company (β_5_), and residual (e_*sdi*_). Since within-person variability in performance across assessment sessions is of primary interest, especially in its association with cognitive status, the residual variance was allowed to depend on cognitive status and some additional factors. Specifically, the log of the residual variance is a linear combination of an intercept (*z*_0_), linear and quadratic trends across assessments sessions (*z*_1_, *z*_2_, respectively), linear and quadratic trends across days (*z*_3_, *z*_4_, respectively), cognitive status (*z*_5_), age (*z*_6_), education (*z*_7_), sex (*z*_8_), race/ethnicity (*z*_9_), within-person and between-person distraction (*z*_10_, *z*_11_, respectively), within-person and between-person location (*z*_12_, *z*_13_, respectively), and within-person and between-person social company (*z*_14_, *z*_15_, respectively). At level-2 (day-level), cognitive performance for person *i* on day *d* was defined as a function of an intercept (δ_00i_), linear (δ_01_) and quadratic (δ_02_) trends across days, and a random effect for the intercept to allow for within-person variation across days (U_0di_), of which the variance is allowed to differ between MCI and CU groups. At level-3 (person-level), mean cognitive performance was defined as a function of cognitive status (γ_001_), age (γ_002_), education (γ_003_), sex (γ_004_), race/ethnicity (γ_005_), between-person distraction (γ_006_), between-person location (γ_007_), between-person social company (γ_008_), and a random effect for the intercept to allow for variation across persons (*V*_00i_), of which the variance is allowed to differ between MCI and CU groups.

*Z*-tests were conducted to test the difference in level-2 (day-level) variability in cognitive performance (U_0di_ in Equation 1) between two independent groups, CU and MCI. To compare the estimates of day-level variability for the CU and MCI groups (*b*_1_ and *b*_2_), the test statistic Z was calculated as:


$$\begin{array}{l} Z\;=\;\frac{b_{1}-b_{2}}{\sqrt{\sigma_{1}^{2}+\sigma_{2}^{2}}} \end{array}$$

Where σ_1_ and σ_2_ were the standard errors of *b*_1_ and *b*_2_, respectively.

## Results

### Descriptive Statistics

**Figure 4:**
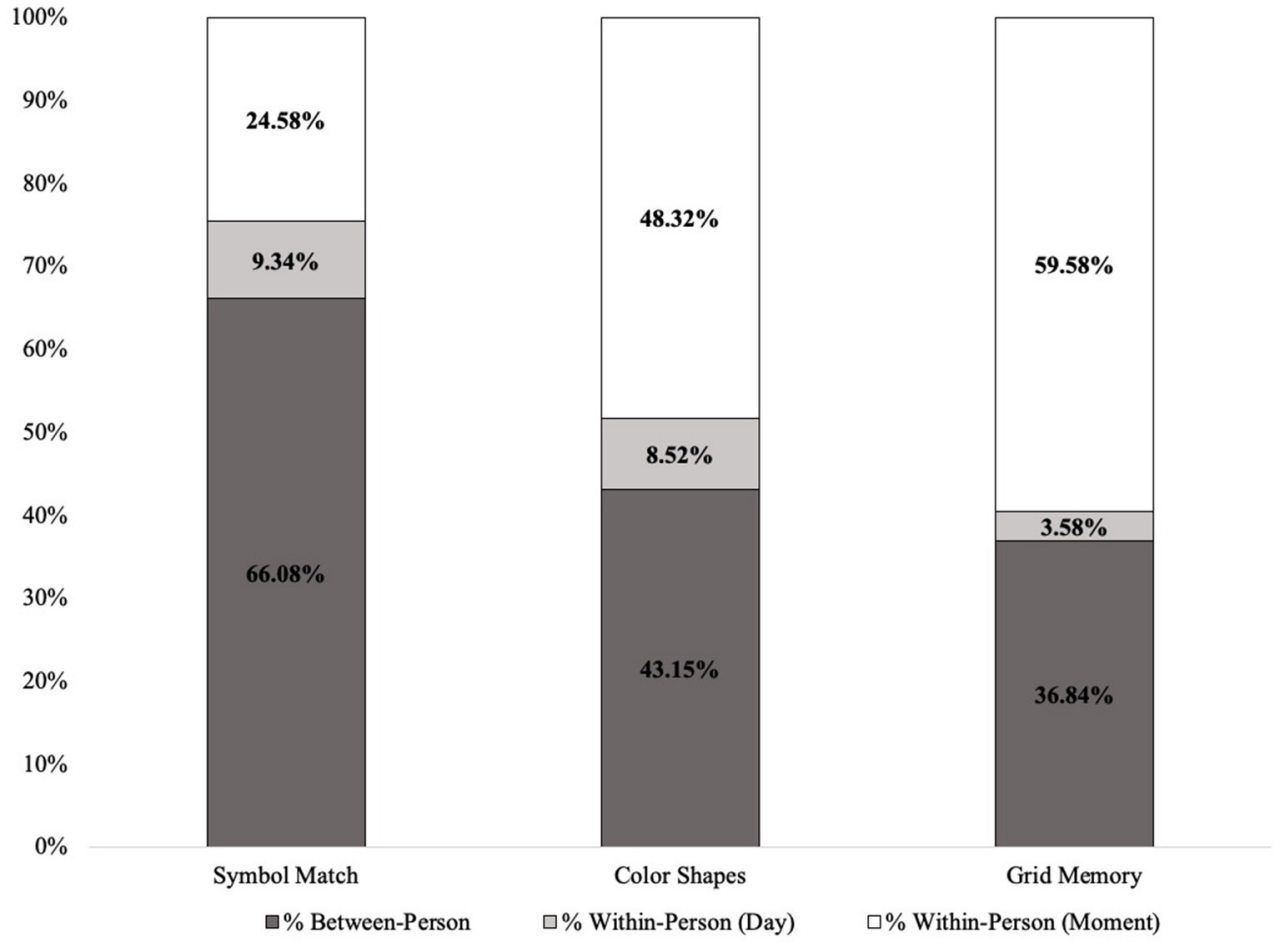
Variance decompositions for mobile cognitive tests.

Table 1 includes all descriptive statistics for mobile cognitive tests, sociodemographic variables, and EMA context variables within the cohort. Compared to participants who were CU, individuals with MCI were significantly older, completed fewer years of education, were more likely to be Non-Hispanic Black and less likely to be Non-Hispanic White, and performed worse on all three mobile cognitive tests (ps < 0.05). Unconditional MLMs showed significant variation in all three mobile cognitive tests (Figure 4). For Symbol Match, 66.1% of the variance reflected between-person differences, with 9.3% of the variance reflecting within-person variation across days, and the remaining 24.6% reflected within-person variation across sessions. For Color Shapes, 43.2% of the variance reflected between-person differences, with 8.5% of the variance reflecting within-person variation across days, and the remaining 48.3% reflected within-person variation across sessions. For Grid Memory, 36.8% of the variance reflected between-person differences, with 3.6% of the variance reflecting within-person variation across days, and the remaining 59.6% reflected within-person variation across sessions.

**Table 1:** Descriptive statistics for sociodemographic variables, contextual variables, and mobile cognitive tests.

	**Full sample (*****N*** **=** **311)**		**CU (*****N*** **=** **211)**		**MCI (*****N*** **=** **100)**		
**Variable**	**Mean (SD)**	**Range**	**Mean (SD)**	**Range**	**Mean (SD)**	**Range**	***P***
Adherence Rate to EMA Protocol	–	–	–	–	–	–	<0.01
Mean adherence (%)	82.90 (16.20)	21.90, 106.20	85.20 (14.20)	21.90, 103.10	78.10 (19.00)	24.00, 106.20	
Median adherence (%)	88.50	75.00, 94.80	89.60	79.20, 94.80	81.80	67.70, 91.70	
Age	77.46 (4.86)	70.40, 90.60	77.06 (4.80)	70.40, 90.60	78.30 (4.90)	70.70, 90.60	<0.05
Education (in years)	14.98 (3.56)	2, 25	15.35 (3.46)	5, 25	14.20 (3.66)	2, 20	<0.01
Female (%)	67.20	–	67.30	–	67.00	–	0.96
Race/ethnicity	–	–	–	–	–	–	–
Non-hispanic white (%)	45.34	–	50.71	–	34.00	–	<0.01
Non-hispanic black (%)	40.19	–	36.49	–	48.00	–	<0.05
Other (%)	14.47	–	12.80	–	18.00	–	0.14
EMA contexts (proportion of assessments)							
Distracted	0.17 (0.16)	0, 0.96	0.17 (0.14)	0, 0.68	0.17 (0.19)	0, 0.96	0.93
Alone	0.44 (0.35)	0, 0.99	0.43 (0.35)	0, 0.97	0.45 (0.36)	0, 0.99	0.63
At home	0.78 (0.16)	0.08, 1.00	0.77 (0.16)	0.08, 0.98	0.80 (0.14)	0.44, 1.00	0.08
Mobile cognitive tests (person means)							
Processing speed: response time (sec)	3.37 (0.90)	1.41, 7.00	3.08 (0.72)	1.87, 5.67	3.96 (0.96)	1.41, 7.00	<0.001
Relational binding: hit/false alarm (%)	0.60 (0.28)	−0.06, 0.98	0.68 (0.24)	−0.06, 0.99	0.43 (0.29)	−0.05, 0.97	<0.001
Spatial working memory: sum of errors	4.62 (1.61)	0.28, 8.31	4.29 (1.59)	0.28, 8.31	5.30 (1.44)	0.87, 8.06	<0.001

*CU = cognitively unimpaired; MCI = Classification of mild cognitive impairment (MCI) defined by Jak/Bondi criteria; Range = full range of data for each variable; P, p-values from evaluation of cognitive status group differences in study variables using chi-square tests for categorical variables and independent t-tests for continuous variables; Median adherence, values under the “Mean (SD)” columns reflect median adherence rates and values under the “Range” columns reflect interquartile ranges. Upper bounds of range values for Mean Adherence (%) exceed 100% because participants were able to self-initiate additional assessment sessions that resulted in some participants completing more than 96 assessments sessions. Other: Category including Hispanic White (9.63%), Hispanic Black (2.94%), Asian (1.28%), other (0.41%), and more than one race (0.40%). Response Time = higher values indicate slower/worse performance in Symbol Match test of processing speed. Hit/False Alarm (%) = higher values indicate better performance in Color Shapes test of visual short-term memory binding. Sum of Errors = higher values indicate worse performance in Grid Memory test of spatial working memory*.

### Heterogenous Variance Models Assessing Cognitive Status Differences in Mean and Variability in Mobile Cognitive Test Performance

#### Mean Performance

**Figure 5:**
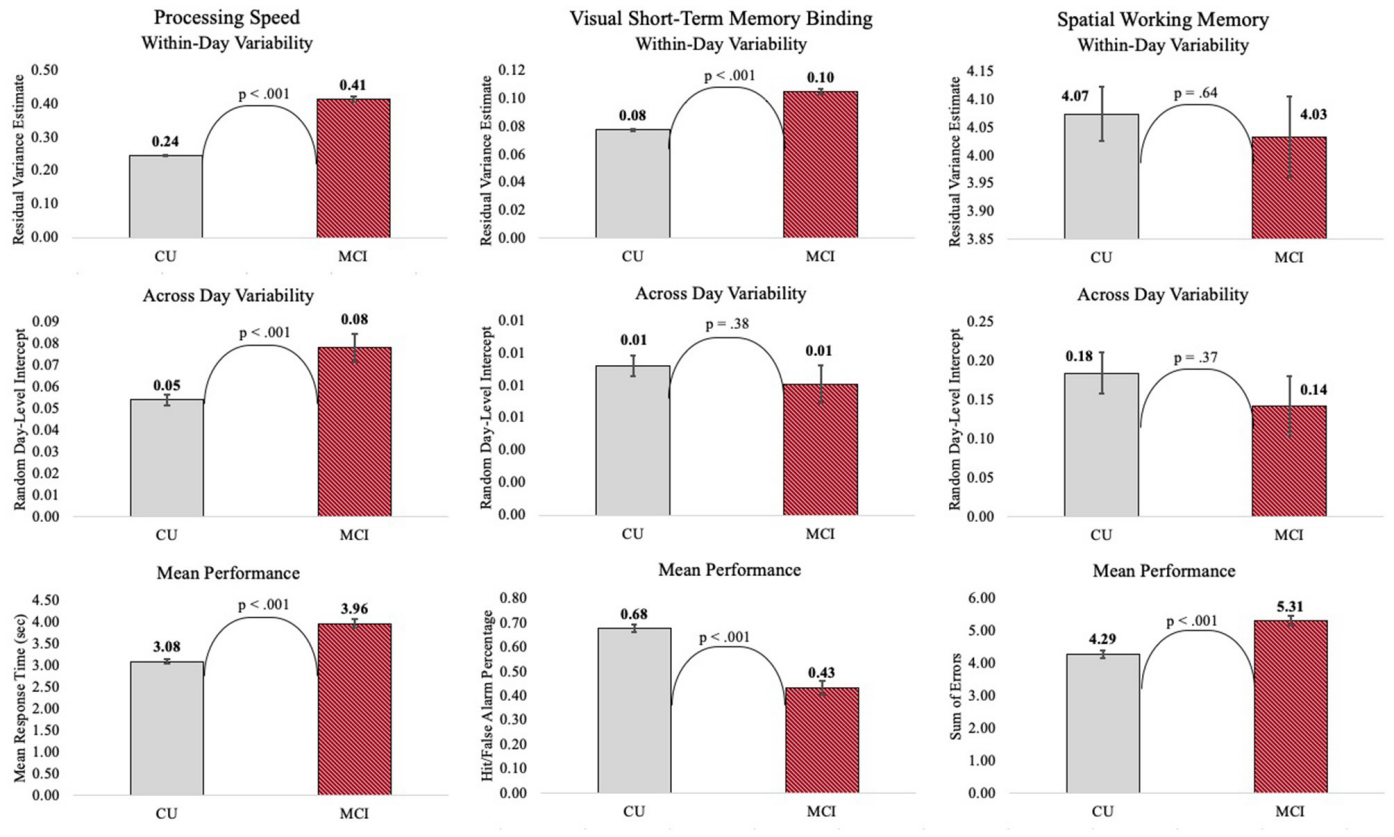
Cognitive status differences in mean and variability in mobile cognitive performance.

Individuals with MCI exhibited significantly worse mean performance on all three mobile cognitive tests (Tables 2–4, Model 1; Figure 5). Compared to participants who were CU, individuals with MCI were 0.88 s slower on the Symbol Match test of processing speed (Est. = 0.88, SE = 0.11, *p* < 0.001), exhibited a 24% lower hit/false alarm percentage score on the Color Shapes test of visual short-term memory binding (Est. = −0.24, SE = 0.03, *p* < 0.001), and committed 1.03 more errors on the Grid Memory test of spatial working memory (Est. = 1.03, SE = 0.18, *p* < 0.001). All associations between MCI status and worse mean performance were robust to adjustment for sociodemographic and EMA contextual variables (see Tables 2–4, Model 2).

**Table 2:** Heterogeneous variance models examining cognitive status differences in mean and variability in symbol match performance.

**Symbol match test of processing speed**		
	**Model 1: unadjusted main effects**	**Model 2: sociodemographic and EMA context adjusted**
**Parameter**	**Estimate (SE)**	**Estimate (SE)**
**Fixed effects**		
Model Mean		
Intercept (γ_001_)	3.57 (0.05)^***^	3.00 (0.25)^***^
Session linear trend (β_1_)	−0.05 (0.01)^***^	−0.001 (0.01)
Session quadratic trend (β_2_)	0.01(0.001)^***^	0.004 (0.003)^†^
Day linear trend (δ_01_)	−0.12 (0.004)^***^	−0.10 (0.01)^***^
Day quadratic trend (δ_02_)	0.01 (0.001)^***^	0.004 (0.001)^***^
Cognitive status (0 = CU, 1 = MCI)	0.88 (0.11)^***^	0.79 (0.11)^***^
(γ_001_)		
Age (centered at mean 77 years)		0.02 (0.01)^*^
(γ_002_)		
Education (centered at mean 15		−0.02 (0.01)^†^
years) (γ_003_)		
Sex (0 = Male, 1 = Female) (γ_004_)		−0.07 (0.10)
Race/ethnicity–non-hispanic black		0.17 (0.10)^†^
(ref: non-hispanic white) (γ_005_)		
Race/ethnicity–other (ref:		0.19 (0.14)
non-hispanic white) (γ_005_)		
Within-person distracted (β_3_)		0.11 (0.01)^***^
Within-person alone (β_5_)		−0.01 (0.01)
Within-person home (β_4_)		0.02 (0.01)
Between-person distracted (γ_006_)		0.35 (0.30)
Between-person alone (γ_008_)		0.04 (0.13)
Between-person home (γ_007_)		0.41 (0.28)
Model within-person variability (residual variance)		
Exp(Intercept) (Exp(*z*_0_))	0.42 (0.01)^***^	0.39 (0.03)^***^
Session linear trend (*z*_1_)	−0.09 (0.02)^***^	−0.11 (0.03)^***^
Session quadratic trend (*z*_2_)	0.02 (0.003)^***^	0.02 (0.01)^**^
Day linear trend (*z*_3_)	−0.12 (0.01)^***^	−0.12 (0.01)^***^
Day quadratic trend (*z*_4_)	0.01 (0.001)^***^	0.01 (0.001)^***^
Cognitive status (0 = CU, 1 = MCI)	0.53 (0.02)^***^	0.52 (0.03)^***^
(*z*_5_)		
Age (centered at mean 77 years)		0.001 (0.003)
(*z*_6_)		
Education (centered at mean 15		−0.03 (0.004)^***^
years) (*z*_7_)		
Sex (0 = Male, 1 = Female) (*z*_8_)		0.03 (0.03)
Race/ethnicity–non-hispanic black		0.19 (0.03)^***^
(ref: non-hispanic white) (*z*_9_)		
Race/ethnicity–other (ref:		0.12 (0.04)^**^
non-hispanic white) (*z*_9_)		
Within-person distracted (*z*_10_)		0.20 (0.04)^***^
Within-person alone (*z*_14_)		−0.10 (0.04)^**^
Within-person home (*z*_12_)		−0.12 (0.04)^***^
Between-person distracted (*z*_11_)		−0.13 (0.09)
Between-person alone (*z*_15_)		−0.09 (0.05)^†^
Between-person home (*z*_13_)		0.15 (0.09)^†^
**Random effects**		
Level-3 (Person) (*V*_00i_)		
Intercept (CU)	0.50 (0.05)^***^	0.45 (0.05)^***^
Intercept (MCI)	0.89 (0.13)^***^	0.86 (0.13)^***^
Level-2 (Day) (*U*_0di_)		
Intercept (CU)	0.04 (0.003)^***^	0.04 (0.003)^***^
Intercept (MCI)	0.06 (0.01)^***^	0.05 (0.01)^***^
−2LL	43745.0	28203.8

*N = 311. ref = reference group; CU = cognitively unimpaired; Cognitive status, Classification of mild cognitive impairment (MCI) scored as 0 = CU, 1 = MCI. The level-2 and level-3 random intercepts are allowed to have different variance between CU and MCI participants. Parameters under Model Within-Person Variability (Residual Variance) = fixed effects predicting variability in cognitive performance across assessment sessions using log-linear prediction of the residual variance. Coefficients from Equation (1) appended to parameter labels to align table contents with the analytic plan*.

† *p < 0.10*.

* *p < 0.05*.

** *p < 0.01*.

*** *p < 0.001*.

**Table 3:** Heterogeneous variance models examining cognitive status differences in mean and variability in color shapes performance.

**Color shapes test of visual short-term memory binding**		
	**Model 1: unadjusted main effects**	**Model 2: sociodemographic and context adjusted**
**Parameter**	**Estimate (SE)**	**Estimate (SE)**
**Fixed effects**		
Model mean		
Intercept (γ_001_)	0.50 (0.02)^***^	0.49 (0.08)^***^
Session linear trend (β_1_)	0.004 (0.003)	0.002 (0.01)
Session quadratic trend (β_2_)	−0.001 (0.001)	−0.001 (0.001)
Day linear trend (δ_01_)	0.04 (0.002)^***^	0.03 (0.002)^***^
Day quadratic trend (δ_02_)	−0.002 (0.001)^***^	−0.001 (0.001)^***^
Cognitive status (0 = CU, 1 = MCI)	−0.24 (0.03)^***^	−0.21 (0.03)^***^
(γ_001_)		
Age (centered at mean 77 years)		−0.01 (0.003)^*^
(γ_002_)		
Education (centered at mean 15		0.02 (0.004)^***^
years) (γ_003_)		
Sex (0 = Male, 1 = Female) (γ_004_)		−0.01 (0.03)
Race/ethnicity–non-hispanic black		−0.08 (0.03)^*^
(ref: non-hispanic white) (γ_005_)		
Race/ethnicity–other (ref:		0.004 (0.04)
non-hispanic white) (γ_005_)		
Within-person distracted (β_3_)		−0.04 (0.01)^***^
Within-person alone (β_5_)		0.003 (0.01)
Within-person home (β_4_)		0.04 (0.01)^***^
Between-person distracted (γ_006_)		0.02 (0.09)
Between-person alone (γ_008_)		0.05 (0.04)
Between-person home (γ_007_)		0.01 (0.09)
Model within-person variability (residual variance)		
Exp(Intercept) (Exp(*z*_0_))	0.12 (0.004)^***^	0.14 (0.01)^***^
Session linear trend (*z*_1_)	−0.02 (0.02)	0.01 (0.03)
Session quadratic trend (*z*_2_)	−0.001 (0.003)	−0.01 (0.01)
Day linear trend (*z*_3_)	−0.11 (0.01)^***^	−0.12 (0.01)^***^
Day quadratic trend (*z*_4_)	0.01 (0.001)^***^	0.01 (0.001)^***^
Cognitive status (0 = CU, 1 = MCI)	0.32 (0.02)^***^	0.26 (0.03)^***^
(*z*_5_)		
Age (centered at mean 77 years)		0.02 (0.003)^***^
(*z*_6_)		
Education (centered at mean 15		−0.02 (0.03)
years) (*z*_7_)		
Sex (0 = Male, 1 = Female)		0.14 (0.03)^***^
(*z*_8_)		
Race/ethnicity–non-hispanic black		0.13 (0.03)^***^
(ref: non-hispanic white) (*z*_9_)		
Race/ethnicity–other (ref:		0.18 (0.04)^***^
non-hispanic white) (*z*_9_)		
Within-person distracted (*z*_10_)		0.21 (0.03)^***^
Within-person alone (*z*_14_)		−0.02 (0.03)
Within-person home (*z*_12_)		−0.06 (0.03)^†^
Between-person distracted (*z*_11_)		−0.03 (0.09)
Between-person alone (*z*_15_)		−0.24 (0.05)^**^
Between-person home (*z*_13_)		−0.05 (0.09)
**Random effects**		
Level-3 (Person) (*V*_00i_)		
Intercept (CU)	0.06 (0.01)^***^	0.05 (0.01)^***^
Intercept (MCI)	0.08 (0.01)^***^	0.08 (0.01)^***^
Level-2 (Day) (*U*_0di_)		
Intercept (CU)	0.01 (0.01)^***^	0.01 (0.001)^***^
Intercept (MCI)	0.01 (0.01)^***^	0.01 (0.001)^***^
−2LL	12035.0	7533.2

*N = 311. ref = reference group; CU = cognitively unimpaired; Cognitive status, Classification of mild cognitive impairment (MCI) scored as 0 = CU, 1 = MCI. The level-2 and level-3 random intercepts are allowed to have different variance between CU and MCI participants. Parameters under Model Within-Person Variability (Residual Variance) = fixed effects predicting variability in cognitive performance across assessment sessions using log-linear prediction of the residual variance. Coefficients from Equation (1) appended to parameter labels to align table contents with the analytic plan*.

† *p < 0.10*.

* *p < 0.05*.

** *p < 0.01*.

*** *p < 0.001*.

**Table 4:** Heterogeneous variance models examining cognitive status differences in mean and variability in grid memory performance.

**Grid memory test of spatial working memory**		
	**Model 1: unadjusted main effects**	**Model 2: sociodemographic and context adjusted**
**Parameter**	**Estimate (SE)**	**Estimate (SE)**
**Fixed effects**		
Model mean		
Intercept (γ_001_)	4.66 (0.12)^***^	3.72 (0.46)^***^
Session linear trend (β_1_)	0.06 (0.02)^**^	0.03 (0.04)
Session quadratic trend (β_2_)	−0.004 (0.004)	0.01 (0.01)
Day linear trend (δ_01_)	−0.08 (0.01)^***^	−0.08 (0.01)^***^
Day quadratic trend (δ_02_)	0.002 (0.001)^*^	0.002 (0.001)^†^
Cognitive status (0 = CU, 1 = MCI)	1.03 (0.18)^***^	0.79 (0.16)^***^
(γ_001_)		
Age (centered at mean 77 years)		−0.01 (0.02)
(γ_002_)		
Education (centered at mean 15		−0.16 (0.02)^***^
years) (γ_003_)		
Sex (0 = Male, 1 = Female) (γ_004_)		0.91 (0.17)^***^
Race/ethnicity–non-hispanic black		0.43 (0.18)^*^
(ref: non-hispanic white) (γ_005_)		
Race/ethnicity–other (ref:		0.06 (0.24)
non-hispanic white) (γ_005_)		
Within-person distracted (β_3_)		0.26 (0.05)^***^
Within-person alone (β_5_)		−0.07 (0.05)
Within-person home (β_4_)		−0.18 (0.05)^***^
Between-person distracted (γ_006_)		0.84 (0.50)^†^
Between-person alone (γ_008_)		−0.06 (0.24)
Between-person home (γ_007_)		0.28 (0.52)
Model within-person variability (residual variance)		
Exp(Intercept) (Exp(*z*_0_))	4.72 (0.14)^***^	4.17 (0.36)^***^
Session linear trend (*z*_1_)	−0.03 (0.02)	−0.04 (0.03)
Session quadratic trend (*z*_2_)	0.004 (0.003)	0.01 (0.01)
Day linear trend (*z*_3_)	−0.03 (0.01)^***^	−0.02 (0.01)^*^
Day quadratic trend (*z*_4_)	0.001 (0.001)^*^	0.001 (0.001)
Cognitive status (0 = CU, 1 = MCI)	−0.01 (0.02)	0.01 (0.03)
(*z*_5_)		
Age (centered at mean 77 years)		−0.003 (0.003)
(*z*_6_)		
Education (centered at mean 15		−0.01 (0.004)
years) (*z*_7_)		
Sex (0 = Male, 1 = Female) (*z*_8_)		0.08 (0.03)^**^
Race/ethnicity–non-hispanic black		−0.01 (0.03)
(ref: non-hispanic white) (*z*_9_)		
Race/ethnicity–other (ref:		−0.003 (0.04)
non-hispanic white) (*z*_9_)		
Within-person distracted (*z*_10_)		0.08 (0.03)^*^
Within-person alone (*z*_14_)		−0.02 (0.03)
Within-person home (*z*_12_)		−0.03 (0.03)
Between-person distracted (*z*_11_)		−0.01 (0.09)
Between-person alone (*z*_15_)		−0.02 (0.03)
Between-person home (*z*_13_)		0.10 (0.09)
**Random effects**		
Level-3 (Person) (*V*_00i_)		
Intercept (CU)	2.48 (0.25)^***^	1.86 (0.19)^***^
Intercept (MCI)	2.00 (0.29)^***^	1.37 (0.22)^***^
Level-2 (Day) (*U*_0di_)		
Intercept (CU)	0.18 (0.03)^***^	0.20 (0.04)^***^
Intercept (MCI)	0.14 (0.04)^***^	0.18 (0.06)^**^
−2LL	107016.1	69955.1

*N = 311. ref = reference group. CU = cognitively unimpaired; Cognitive Status = Classification of mild cognitive impairment (MCI) scored as 0 = CU, 1 = MCI. The level-2 and level-3 random intercepts are allowed to have different variance between CU and MCI participants. Parameters under Model Within-Person Variability (Residual Variance) = fixed effects predicting variability in cognitive performance across assessment sessions using log-linear prediction of the residual variance. Coefficients from Equation (1) appended to parameter labels to align table contents with the analytic plan*.

† *p < 0.10*.

* *p < 0.05*.

** *p < 0.01*.

*** *p < 0.001*.

#### Variability Within-Day Across Assessment Sessions

Individuals with MCI exhibited significantly greater within-day variability on two of the three mobile cognitive tests (Tables 2–4, Model 1; Figure 5). Specifically, compared to CU participants, the residual variance (on log scale) for individuals with MCI was 0.53 units higher in the Symbol Match test of processing speed (Est. = 0.53, SE = 0.02, *p* < 0.001; or 70% higher residual variance among MCI vs. CU), 0.32 units higher in the Color Shapes test of visual short-term memory binding (Est. = 0.32, SE = 0.02, *p* < 0.001; or 38% higher residual variance among MCI vs. CU), but not significantly different in the Grid Memory test of spatial working memory (Est. = −0.01, SE = 0.02, *p* = 0.63). Thus, compared to participants who were CU, individuals with MCI were more variable within-days across assessments in processing speed and visual short-term memory binding performance. Variability in spatial working memory performance did not differ between individuals with MCI and those who were CU.

Associations between MCI status and variability in performance across EMA assessment sessions were robust to adjustment for sociodemographic and EMA contextual variables (see Tables 2–4; Model 2). Compared to CU participants, the residual variance (on log scale) for individuals with MCI was 0.52 units higher in the Symbol Match test of processing speed (Est. = 0.52, SE = 0.03, *p* < 0.001; or 68% higher residual variance among MCI vs. CU), 0.26 units higher in the Color Shapes test of visual short-term memory binding (Est. = 0.26, SE = 0.03, *p* < 0.001; or 30% higher residual variance among MCI vs. CU), but not significantly different in the Grid Memory test of spatial working memory (Est. = 0.01, SE = 0.03, *p* = 0.61). Thus, MCI differences in the variability in mobile cognitive performance within-days across assessments of processing speed and visual short-term memory binding were not a byproduct of within-person fluctuations or between-person differences due to EMA contextual factors.

#### Variability Across Study Days

The variance of a level-2 (day-level) random intercept was estimated for both CU and MCI participants to examine cognitive status differences in the variability in mobile cognitive performance across study days (in addition to within-day variability modeled at level-1). Individuals with MCI exhibited greater variability across study days on the Symbol Match test of processing speed (Table 2, Model 1: MCI: Est. = 0.06, SE = 0.01, *p* < 0.001; CU: Est. = 0.04, SE = 0.003, *p* < 0.001), but exhibited similar or slightly smaller amounts of day-level variability on the Color Shapes test of visual short-term memory binding (MCI: Est. = 0.01, SE = 0.01, *p* < 0.001; CU: Est. = 0.01, SE = 0.003, *p* < 0.001) and Grid Memory test of spatial working memory (MCI: Est. = 0.14, SE = 0.04, *p* < 0.001; CU: Est. = 0.18, SE = 0.03, *p* < 0.001). Z tests of these point estimates revealed the only statistically significant cognitive status difference in day-level variability was in the Symbol Match test (Z = 2.88, *p* < 0.01). Variability across study days was not significantly different between MCI and CU participants for Color Shapes (*Z* = −0.15, *p* = 0.88) and Grid Memory (*Z* = −0.80, *p* = 0.42) tests.

### EMA Contextual Influences on Within-Day Variability in Mobile Cognitive Performance

#### Within-Person Associations

Within-day variability in performance on all three mobile cognitive tests was higher when participants were distracted vs. when participants were not distracted (Symbol Match: Est. = 0.20, SE = 0.04, *p* < 0.001; Color Shapes: Est. = 0.21, SE = 0.03, *p* < 0.001; Grid Memory: Est. = 0.08, SE = 0.03, *p* < 0.05). Within-day variability in Symbol Match performance was lower when participants were alone vs. when participants were with others (Est. = −0.10, SE = 0.04, *p* < 0.01). No within-person association emerged between social company and variability for Color Shapes (Est. = −0.02, SE = 0.03, *p* = 0.61) and Grid Memory (Est. = −0.02, SE = 0.03, *p* = 0.50). Within-day variability in Symbol Match performance was lower (Est. = −0.12, SE = 0.04, *p* < 0.001) when participants were home vs. when participants were elsewhere. No within-person association emerged between location and variability for Color Shapes (Est. = −0.06, SE = 0.03, *p* = 0.07) and Grid Memory (Est. = −0.03, SE = 0.03, *p* = 0.38).

#### Between-Person Associations

Individual differences in the proportion of distracted sessions were not associated with within-day variability in any of the 3 mobile tests (Symbol Match: Est. = −0.13, SE = 0.09, *p* = 0.15; Color Shapes: Est. = −0.03, SE = 0.09, *p* = 0.72; Grid Memory: Est. = −0.01, SE = 0.09, *p* = 0.87). Individual differences in the proportion of sessions completed alone was associated with Color Shapes performance (Est. = −0.24, SE = 0.05, *p* < 0.001) such that participants who were alone more often had less within-day variability in performance. No between-person association between social company and variability emerged for Symbol Match (Est. = −0.09, SE = 0.05, *p* = 0.06) and Grid Memory (Est. = −0.03, SE = 0.05, *p* = 0.57). Individual differences in the proportion of sessions completed at home was not associated with within-day variability in any of the 3 mobile tests (Symbol Match: Est. = 0.15, SE = 0.09, *p* = 0.09; Color Shapes: Est. = −0.05, SE = 0.09, *p* = 0.55; Grid Memory: Est. = 0.10, SE = 0.09, *p* = 0.26).

## Discussion

In a diverse sample of community-dwelling older adults, we explored the potential utility of variability in mobile cognitive performance as a sensitive marker of MCI. With heterogeneous variance multilevel models, we simultaneously assessed differences in mean performance and within-person variability between CU and MCI groups. We evaluated differences in within-day variability and day-to-day variability in mobile cognitive performance to assess which timescale of variability is most sensitive to MCI. We included mobile cognitive tests of processing speed, visual short-term memory binding, and spatial working memory to ensure broad evaluation of findings across multiple domains of cognitive function. Overall, results suggest variability in cognitive performance distinguishes MCI and exhibits dissociative patterns by timescale and cognitive domain. All findings were robust to adjustment for distraction, location, and social company, providing additional support for the hypothesis that differences in variability between the MCI and CU groups is not a byproduct of within-person and between-person contextual factors. We discuss the findings in comparison and extension of prior research evaluating variability in cognitive performance as a promising indicator of normative and pathological cognitive aging [e.g., (6, 7, 22)]. Considerations for monitoring and identifying individuals at increased risk of cognitive impairment with smartphone-based digital health approaches are discussed in the context of measurement burst designs with EMA protocols.

### Variability in Mobile Cognitive Performance Is Sensitive to MCI

Consistent with expectations and previous work [e.g., (6, 7)], both mean and variability in performance differed between the MCI and CU groups. Compared to older adults who were CU, participants with MCI exhibited worse mean performance on all three mobile cognitive tests–slower average processing speed, worse average visual short-term memory binding performance, and worse average spatial working memory performance. These observed cognitive status differences in level of performance for all tested domains of cognitive function are broadly consistent with extant literature demonstrating MCI-related deficits in multiple domains of cognitive function including processing speed, as well as tests requiring visuospatial skills and general executive functioning [e.g., (6, 54, 55)]. The main advance of the current study, however, is that differences between participants with MCI and those who were CU *also* emerged in variability in cognitive performance under naturalistic circumstances.

Unlike the uniform reductions in mean performance in the MCI group, the cognitive status differences in variability in mobile cognitive performance emerged for two of the three mobile cognitive tests and domains. Consistent with our hypotheses, individuals with MCI exhibited greater within-day variability for mobile tests of processing speed and visual short-term memory binding. The lack of differences on the test of spatial working memory was unexpected; the implications of this finding are discussed below. To our knowledge, the present study is the first examination of cognitive status differences in the variability in mobile cognitive performance across assessment sessions nested within days in naturalistic settings. Thus, our findings extend previous work in two primary ways. First, the extant literature on cognitive status differences in variability in cognitive performance largely operationalize variability as trial-level fluctuations in speeded responses [e.g., (20)]. We demonstrate that variability from assessment-to-assessment within a day, a longer timescale than trial-to-trial fluctuations, is also sensitive to MCI. The present study's findings are broadly consistent with expectations formulated based on trial-level variability's established sensitivity to MCI [e.g., (6, 7)] and links to central nervous system integrity [e.g., (24)]. Though prior research has shown that memory binding tasks are sensitive to ADRD (32, 33, 36), past work assessed level of performance but not variability. Our findings on variability in performance suggest that individuals with MCI may have compromised neurological capacity to consistently perform tasks throughout the day that require speeded responses and memory binding. Our work also helps address an open question in the field as to whether mobile cognitive tests within an EMA protocol can be used to evaluate cognitive status differences in variability in mobile cognitive performance across assessments. We provide preliminary evidence for this smartphone-based digital health approach with findings that suggest individuals with MCI exhibit greater variability in mobile cognitive test performance compared to those were CU.

These EMA-based findings for within-day variability in performance on smartphones are broadly consistent with prior work in laboratory settings that has found trial-level variability to be associated with greater likelihood of MCI classification at cross-section (6) and greater odds of CIND classification 4 years later (7). Thus, the present study extends prior research with preliminary evidence suggesting that assessments of variability over longer timescales than the trial-level also show promise for identifying early clinical manifestations of cognitive impairment. Longitudinal work is needed however, to fully evaluate its predictive validity in detecting incident MCI and other pathological cognitive aging outcomes years later.

Individuals with MCI and participants who were CU did not differ in variability on our test of spatial working memory. The dissociative pattern among tests supports and extends conceptual and empirical accounts demonstrating that mean and variability reflect distinct cognitive processes [e.g., (7, 16, 56, 57)]. If mean and variability conferred redundant information, we would have expected that the tests that show mean differences would also show cognitive status differences in variability. While worse mean performance in general (i.e., across all three mobile tests) appears to be a ubiquitous marker of MCI, variability in processing speed and visual short-term memory binding performance may provide specific detection of MCI. Variability in tasks without speeded responses or memory binding paradigms, in contrast, may have less utility in identifying those at increased risk of MCI. A dissociative pattern could also arise if the Grid Memory test provides a less reliable estimate of variability. Future research leveraging longitudinal data across multiple years is needed to assess the separate and joint predictive validity of baseline mean and variability (and rates of change in mean and variability) for assessing cognitive decline [e.g., (22)] and MCI [e.g., (7)] years later. To determine if the dissociation in mean performance and variability in performance reflects the domain being measured or an attribute of the test, additional exploration with a variety of domain-specific measures would be required.

### Value of EMA Measurement Burst Design for Smartphone-Based Digital Health Approach to Assessing Risk of Cognitive Impairment

In utilizing a digital health approach and leveraging the EMA measurement burst design of EAS, we were able to operationalize multiple timescales of cognitive performance variability in naturalistic settings. The innovative integration of smartphones equipped with ultra-brief cognitive tests and multiple assessments repeated every day for 16 consecutive days allowed for evaluation of within-day and day-to-day variability–relatively untapped timescales of variability as possible markers of MCI. We would not have been able to evaluate these timescales of short-term variability without the EMA measurement burst design and digital health approach. Distinguishing within-day variability from day-to-day variability revealed more pronounced cognitive status differences in within-day variability compared to day-to-day variability. Compared to CU participants, individuals with MCI exhibited significantly greater within-day variability in mobile tests of processing speed and visual short-term memory binding. These cognitive status differences in variability were less pronounced at the day-to-day timescale for the tests of processing speed and visual short-term memory binding, and were ultimately non-significant for the test of visual short-term memory binding.

Our results may have important implications for the design of future digital health approaches to measuring mobile cognitive performance and cognitive status differences therein. Given within-day variability appears to be more sensitive to MCI than day-to-day variability in the current study, measurement burst designs with EMA protocols and research questions focused on differentiating cognitive status may consider prioritizing more assessments within a given study day rather than adding additional study days in each measurement burst epoch. This is an important implication for EMA protocol design because balancing sampling density with participant burden is essential for ensuring adherence to the protocol while retaining capacity to observe systematic variability in cognitive performance that is sensitive to MCI.

Our digital health approach was situated within a tradition of intraindividual variability (IIV) approaches that harness intensive repeated measurements (e.g., EMA) and technological advancements for capturing meaningful short-term variation and enhanced sensitivity of behavioral markers of impairment [(58, 59) for a review on EMA, see (60); for reviews on IIV approaches, see (16, 61, 62)]. While the present study identified variability in mobile cognitive performance across assessment sessions as a behavioral marker of MCI, other indices of behavioral variability show promise as markers of impairment as well. Indeed, past research adopting IIV approaches have identified a broad range of behavioral markers of cognitive decline and impairment, including variability in gait performance measured by passive in-home infrared sensors (63) and computerized walkways [e.g., (64)], as well as variability in computer mouse movements measured during in-home computer use (65). Each of these behavioral markers lend support for digital health and IIV approaches that assess performance dynamics in naturalistic settings (e.g., EMA protocols, passive monitoring in homes) for sensitive detection of subtle changes within persons and early clinical manifestations of cognitive impairment.

Further, collecting information on the context in which participants perform the mobile cognitive tests helps to specify the moments when variability in performance is heightened or lessened. The current study found within-day cognitive performance variability to be higher when participants were distracted and lower when participants were alone and at home. Additionally, adjusting for EMA context variables when examining cognitive status differences in variability in mobile cognitive performance helps to strengthen the case for variability's sensitivity to MCI if the effects are robust to their inclusion as they were in the current study.

### Limitations and Future Directions

Several limitations of this study should be considered. Analyses were correlational and cross-sectional, which prevent any causal or temporal conclusions concerning the predictive validity of variability as a sensitive indicator of MCI status. Future work is needed to assess the predictive validity of variability in cognitive performance with longitudinal data to determine if greater variability in performance predicts changes in cognitive function and higher odds of incident MCI year(s) later. Importantly, data from bursts 2 and 3 of the EAS EMA measurement burst protocol are currently being processed by the EAS team and will offer an ideal analytic opportunity in future work to assess variability at burst 1 as a predictor of incident MCI at subsequent bursts (i.e., 1–2 years later), as well as other longitudinal research questions pertaining to annual change in variability in cognitive performance within- and between-persons. Further, we cannot rule out the potential for false positive MCI classifications due to the unstable nature of MCI as an indicator of cognitive pathology in general and the use of Jak/Bondi algorithmic criteria of global neuropsychological test performance from a single visit to the EAS clinic. While outside the scope of the present study, longitudinal work with at least a second comprehensive neuropsychological evaluation 1 year later will help to ensure true positive MCI cases and identify instances of reversion in individuals initially classified with MCI.

Finally, the present study examined estimates of variability in performance across assessments, controlling for between-person and within-person contextual influences (distraction, location, social company). It did not evaluate possible between-person and within-person conceptual/theoretical influences on these estimates of variability. Recent work has demonstrated that trial-level variability is systematically related to daily variations in stress (15), negative affect (19), and perceived control (66). In addition to work on trial-level variability, prior work has also found that working memory performance is worse on high-stress days compared to low-stress days (53), as well as days with higher levels of negative affect (67) and anticipatory stress upon waking (14). This line of research broadly informs personalized approaches to optimize cognitive health (68, 69) by characterizing for whom and at what moments modifiable risk factors are related to early indicators of normative and pathological cognitive aging (i.e., indices of variability). With the present study as a foundation, future research should evaluate the extent to which variability in mobile cognitive test performance is impacted by fluctuations in time-varying within-person psychosocial/health factors (e.g., stress, affect, control beliefs, pain, sleep) and individual differences in between-person risk factors (presence of chronic pain, presence of chronic stress, depression diagnosis, diabetes status) and ADRD biomarkers such as β amyloid deposition, pathologic tau, and neurodegeneration [AT[N]; (70)]. Pinpointing within-person and between-person sources of variability (and potential moderating roles of cognitive status) will further clarify the unique utility of variability in cognitive performance as an early marker of cognitive impairment.

Our smartphone-based digital health approach leveraged the innovative platform Mobile Monitoring of Cognitive Change, or M2C2. Part of the larger NIH Mobile Toolbox project, the M2C2 platform is currently in the development, validation, and norming phase with testing underway across a wide range of samples and study designs [e.g., (27, 31)]. See www.mobiletoolbox.org for additional information about the forthcoming NIH Mobile Toolbox.

## Conclusion

Our results demonstrate that a smartphone-based digital health approach to ambulatory assessment of cognitive performance in older adults has the capacity to differentiate individuals with MCI from those who are CU. Results suggest that variability in performance on mobile devices uniquely distinguishes MCI and exhibits dissociative patterns by timescale of variability and cognitive domain. Within-day variability in processing speed and visual short-term memory binding performance may provide specific detection of MCI. The 16-day smartphone-based EMA measurement burst offers novel opportunities to leverage digital technology and performance variability across frequent assessments for studying cognitive function and identifying early clinical manifestations of cognitive impairment.

## Data Availability Statement

The data analyzed in this study is subject to the following licenses/restrictions: Interested collaborators are asked to complete a concept proposal form (details for potential project, paper, or abstract) to be reviewed and forwarded to the Einstein Aging Study Steering Committee for consideration. Requests to access these datasets should be directed to Mindy Katz, MPH at mindy.katz@einsteinmed.org. For additional information on data sharing requests for the Einstein Aging Study, see https://einsteinmed.org/departments/neurology/clinical-research-program/eas/data-sharing.aspx.

## Ethics Statement

The studies involving human participants were reviewed and approved by the Institutional Review Board at Albert Einstein College of Medicine. The patients/participants provided their written informed consent to participate in this study.

## Author Contributions

EC and MS conceived of the presented idea. MK, RL, MS, CD, CW, NR, JH, and JGH contributed to the design and implementation of the Einstein Aging Study protocol and data collection. EC, JQ, CW, and QG performed the statistical analysis. EC wrote the first draft of the manuscript. All authors contributed to manuscript revision, read, and approved the submitted version.

## Funding

This work was supported in part by the National Institute on Aging under Grants P01AG003949, U2CAG060408, and T32AG049676 to Pennsylvania State University.

## Conflict of Interest

The authors declare that the research was conducted in the absence of any commercial or financial relationships that could be construed as a potential conflict of interest.

## Publisher's Note

All claims expressed in this article are solely those of the authors and do not necessarily represent those of their affiliated organizations, or those of the publisher, the editors and the reviewers. Any product that may be evaluated in this article, or claim that may be made by its manufacturer, is not guaranteed or endorsed by the publisher.
